# Reducing Social Media Use Decreases Depression Symptoms: A Meta-Analysis of Randomised Controlled Trials

**DOI:** 10.3390/ejihpe15110222

**Published:** 2025-10-27

**Authors:** Willem May, John M. Malouff, Jai Meynadier

**Affiliations:** School of Psychology, University of New England, Armidale, NSW 2351, Australia; willmay@y7mail.com (W.M.); jmeynad2@une.edu.au (J.M.)

**Keywords:** depression, effects, meta-analysis, social media, reducing use

## Abstract

The association between social media use and depression found in correlational research has prompted widespread concern regarding the consequences of social media use. In response to this evidence, experimental interventions have been used to evaluate whether lowering social media use affects depression. This meta-analysis synthesised results of 10 randomized controlled trials (*N* = 1491) to assess the effect of limiting or refraining from social media use on severity of depressive symptoms. Studies were included if they were randomized control trials involving reducing or eliminating use of social media for a period of time. The results indicate that reducing social media use significantly decreases depressive symptoms, with an effect size of *g* = 0.25, 95% CI [0.10, 0.41], *p* < 0.001, after adjusting for publication bias. Significant heterogeneity was found between studies, with I^2^ indicating that 47% of the variability in effect sizes across studies was due to heterogeneity of true effect size rather than random error. Although interventions aimed at reducing use of social media had twice the depression effect size of interventions aimed at abstinence from social media, the difference was not significant. Two other potential moderators of effect size, intervention length and number of social networks included, were also nonsignificant. Overall, this meta-analysis suggests that limiting social media use is an effective way to reduce symptoms of depression. However, more studies with good research methods are needed to evaluate this conclusion. Also, further research is needed to evaluate long-term effects of limiting or refraining from social media use.

## 1. Introduction

*Social media* is a term used to describe the wide array of web-based platforms and social networking sites that allow users to create and exchange content (Aichner et al., 2021). These platforms enable easy and instantaneous communication between individuals with an internet connection (Cunningham et al., 2021). It is estimated that over five billion people have at least one social media account and that individuals with access to the internet spend an average of 143 min a day using social media (Dixon, 2024; Petrosyan, 2024).

Researchers have expressed concern regarding the extent social media has become entrenched in everyday life and how this may affect us (Yoon et al., 2019). Early investigations into online communication indicate that social networking sites can serve important interpersonal and relational functions for users (Bryant et al., 2011). Social media use has been found to benefit individuals by enhancing well-being (Marciano et al., 2024) and cognitive function (Myhre et al., 2017), while also providing social support (Ellison et al., 2011; Patel et al., 2015; Primack & Escobar-Viera, 2017). However, social networking sites have been scrutinised for their potential to promote negative social comparisons (Samra et al., 2022), bullying (Craig, 2020), addiction (Anderson et al., 2017; Huang, 2020), and eating disorders (Dopelt & Houminer-Klepar, 2025), and their potential to decrease in-person social interactions (Vidal et al., 2020). Despite a wide range of findings across differing populations, research findings overall suggests that l social media use is linked to increased symptoms of low self-esteem and depression (Cunningham et al., 2021; Saiphoo et al., 2020; Ulvi et al., 2022).

A causal relationship between social media use and depression is particularly concerning (Cunningham et al., 2021). Depression is the leading global cause of disability, and over 300 million people suffer from a depressive disorder (World Health Organization, 2017). The disorder correlates with all-cause mortality (Gump et al., 2005), and major depressive disorder is projected to be the largest single contributor to global disease burden by 2030 (World Health Organization, 2011). Symptoms of depression are characterised by feelings of low mood, reduced energy, and loss of enjoyment. These problems are often associated with a range of physical, emotional, and cognitive impairments (Cui, 2015). People who suffer from depression typically first develop symptoms in adolescence or young adulthood (Petito et al., 2020). The incidence of depressive disorders has been steadily rising in this age cohort (Australian Institute of Health and Welfare, 2021; Twenge et al., 2018), and 18- to 24-year-olds are amongst the highest users of social media (Smith & Anderson, 2018).

### 1.1. Correlational Research

A significant, small correlation between time spent using social media and depression was found in various meta-analyses (Cunningham et al., 2021; Ghai et al., 2023; Hancock et al., 2022; Ivie et al., 2020; Vahedi & Zannella, 2019; Vesal & Rahimi, 2021; Yoon et al., 2019). These reviews reported a small positive correlation that ranged from *r* = 0.11 (*p* < 0.001) to *r* = 0.17 (*p* < 0.001). Liu et al. (2022) also conducted a dose–response meta-analysis which found that the likelihood of adolescents experiencing symptoms of depression increased by 13% for each additional hour spent using social media per day (*p* < 0.001).

The strength and direction of the correlation between social media use and depression is likely dependent on how social media is used and the quality of the social media environment (Seabrook et al., 2016). Seabrook et al. (2016) conducted a systematic review that found experiencing positive interaction, social support, or social connectedness consistently correlated with lower levels of depression, while negative interactions and social comparison were consistently correlated with higher levels of depression. Keles et al. (2020) reported in a systematic review that sleep-quality, perceived social support, and rumination often mediated the association between social media use and depression.

Problematic social media use occurs when maladaptive or excessive use produces the behavioural and psychological symptoms of addiction (Bányai et al., 2017; Sun & Zhang, 2020). A meta-analysis by Yigiter et al. (2023) found that the association between depression and social media use was strong for problematic users (*r* = 0.32, *p* < 0.001). An estimated 17% of all users have a problematic relationship with social media (Karlsson et al., 2019; Plackett et al., 2023). These individuals often experience a compulsion to use social networking platforms strong enough to diminish their ability to self-regulate (Reinecke et al., 2022). A reduced level of functioning negatively impacts their work, health, and relationships (Henzel & Håkansson, 2021; Sun & Zhang, 2020).

Fruehwirth et al. (2024) used a longitudinal research method to examine the connection between social media use and various mental health problems. The study found evidence of social media use being connected to later levels of depression. These results buttress the results of cross-sectional correlational studies.

The robust evidence of an association between social media use and depression might suggest that social media use can contribute to depression, although correlation does not show causation. The possibility exists that depressive symptoms caused by unrelated issues could lead to increased social media use (Hartanto et al., 2021). Kardefelt-Winther’s (2014) theory of compensatory internet use proposes that people may use social media to alleviate negative feelings or fulfil unmet psychosocial needs. This theory suggests that individuals suffering from depression may increase their social media use in search of social validation or as an escape from real life issues.

If social media use does cause depression in some individuals, the mechanism could involve fear of missing out (FOMO), negative social comparisons, and negative emotions from efforts to self-regulate. The mechanisms involving FOMO and self-regulation stress might be made less potent by reducing use rather than by eliminating it.

### 1.2. Experimental Research

Tromholt (2016) found that fully abstaining from Facebook for 1-week significantly reduced depressive symptoms and improved affect. The current body of experimental research has focused on emulating this research design by testing interventions that require participants to reduce their social media use (Plackett et al., 2023). These interventions are designed to provide direct evidence on whether reducing social media use causes a reduction in the severity of depression.

A systematic review conducted by Plackett et al. (2023) identified seven randomized controlled trials (RCTs) that measured the effect reducing social media use had on depression. They found that five out of the seven RCTs significantly reduced the severity of depressive symptoms. They concluded that these interventions could be placed in two distinct categories: full abstinence interventions and limited use interventions. Full abstinence interventions require the experimental group to completely forgo the use of at least one social media platform. Limited use interventions require the experimental group to reduce their total time spent using specific or all social media platforms but still allow for some amount of use. Results seem mixed for the two types of intervention, e.g., with de Hesselle and Montag (2024) finding no positive effect for a total-abstinence intervention and Chlupová and Lukavská (2023) finding a significant positive effect for a reduction intervention.

When completed as part of an RCT, interventions can be assessed against a control group that use social networking sites as normal. Possible moderators of effect size include type of intervention (abstinence vs. reduction), intervention length, and number of social networks targeted.

Ramadhan et al. (2024) conducted the only meta-analysis that assessed the effect reducing social media use has on depression, as well as other outcomes. The researchers included three randomised controlled trials and found that full abstinence from social media significantly reduced level of depression. One of the three included studies (Reed et al., 2023) reported only baseline depression levels, and the meta-analysis used those as post-intervention scores. Hence, the depression results of the meta-analysis are not convincing.

### 1.3. Aims of the Present Meta-Analysis

It remains to be seen whether reducing or refraining from social media use leads to less depression. A meta-analysis of a sizeable number of randomized control trials on the topic could provide useful information. We therefore conducted a meta-analysis of RCTs that measured the effect that reducing social media use had on depression. The main aim was to determine whether reducing or refraining from social media use would decrease levels of depression. We hypothesised that limited-use interventions would be more effective than full-abstinence interventions in reducing depressive symptoms. This prediction was made because there are positive social effects of using social media that would be removed by full-abstinence interventions (e.g., Myhre et al., 2017). Also, for adherence and for positive effects on depression, participant autonomy could be important, as it is one of three motivating components of Self-Determination Theory (Deci & Ryan, 2012). We also hypothesised that the longer the intervention and the more social networks it targeted, the more effective it would be in reducing the severity of depressive symptoms. We had no basis for these latter two moderator hypotheses other than the general idea that the more comprehensive an intervention, the more likely it is to have a positive effect.

## 2. Methods

### 2.1. Protocol and Registration

This meta-analysis was completed in accordance with the Preferred Reporting Items for Systematic Reviews and Meta-Analyses (PRISMA) guidelines (Page et al., 2021). The protocol for this study was registered with PROSPERO, https://www.crd.york.ac.uk/prospero/display_record.php?RecordID=531956 (accessed on 1 June 2024).

### 2.2. Eligibility Criteria

Studies were eligible for inclusion in this meta-analysis if they: (a) conducted an intervention that explicitly aimed to reduce (or end) social media use, (b) did not conduct any other intervention in conjunction with social media use reduction, (c) compared the intervention group to a control group with randomised group allocation (RCT study design), (d) assessed pre and post level of depression in both the intervention and control groups using a reliable and valid measure of depression, (e) provided the prerequisite data for effect size calculation. No studies were excluded based on language, date of publication or sample characteristics. Only RCTs were included as they are the gold-standard for evaluating causal relationships and the efficacy of interventions (Hariton & Locascio, 2018).

### 2.3. Information Sources

Eligible studies were identified from multiple sources. A systematic search of the electronic databases ProQuest, PubMed, Scopus and EBSCOhost was conducted. We reviewed the reference list of each identified study and of a previous review (Plackett et al., 2023). Forward citation searching was completed for these studies using Google Scholar. The search for eligible studies concluded in September 2024.

### 2.4. Search Strategy

We searched the titles and abstracts of all studies found in each electronic database using the following search terms: (Facebook OR Twitter OR Instagram OR WhatsApp OR YouTube OR Twitter OR Reddit OR TikTok OR Snapchat OR WeChat OR Weibo OR “social media” OR “online social network*” OR “social network* site*”) AND (Depress*) AND (“Randomised controlled trial” OR “Randomized controlled trial” OR RCT OR Random* OR Trial* OR Experiment* OR Intervention) AND (Addict* OR Excessi* OR Restrict* OR Quit* OR Withdraw* OR Abstin* OR Abstain OR Treat* OR Reduc* OR Eliminat*).

### 2.5. Study Selection Process

Two researchers independently completed the systematic search of electronic databases using the selected search terms. We ensured the accuracy of the search by comparing the total number of results from each search. All disagreements were discussed until a mutual consensus was reached. The eligibility of these studies was then assessed using web-based review management software called Covidence (Veritas Health Innovation, 2024). Both researchers used this software independently to screen the title and abstracts, followed by the full text of the articles. Any disagreements regarding the eligibility of a study were resolved by discussion.

The following data was coded regarding effect size: study author and publication year, pre- and post-intervention means and standard deviations for experimental and control groups, experimental and control group final sample size, pre-post correlation and effect direction. Thai et al. (2021) did not provide pre- and post-intervention data; instead an *F* value and sample size was coded to calculate effect size. The following descriptive data was coded for each study: overall sample size, average participant age, female percentage, the country the study was conducted in, intervention type, duration of the intervention in days, duration from baseline assessment to final assessment in days, number of social media platforms targeted by the intervention.

If a study had multiple experimental groups, data was coded for the experimental group that only required participants to reduce or refrain from social media use. We did not include analyses of interventions that combined social media reduction or refraining from use *and* another intervention. When a study did not publish the necessary data to code the above items, we contacted the author of the study to obtain the missing information or raw data files. All data conversions were completed using formulas from the Cochrane Handbook for Systematic Reviews of Interventions (Li et al., 2024). An example of this was that we used the standard error and sample size statistics reported in Faulhaber et al. (2023) to calculate standard deviation. If the necessary data to calculate effect size could not be coded, the study was excluded from analysis.

If a relevant study report lacked information needed for the meta-analysis, we wrote to the corresponding author and asked for the needed information. In one case, de Hesselle and Montag (2024), we obtained the data file and used that to calculate effects on depression. We used the two depression items and excluded the anxiety items.

### 2.6. Moderator Selection and Coding Process

We coded a total of three moderator variables in this meta-analysis. Each of these variables was present in all studies, demonstrated variance, and could inform the development of future interventions if found to be significant. The first was intervention type, which was coded as either full abstinence or limited use. The second was length of intervention, which was coded as the number of days the experimental group members were required to reduce their social media use. The third was total number of social networking sites targeted.

### 2.7. Study-Method Risk Assessment

Liebherz et al. (2016) found that most standardised quality assessment checklists lack vital criteria or include items that lack relevance, and they suggested researchers create their own specific criteria set to ensure that each item is suitable. We used the Cochrane Collaboration’s tool for assessing risk of bias in randomised trials (Higgins et al., 2011) to design a bias-risk assessment checklist that evaluated the internal validity of each study. The assessment criteria were: (a) whether the sample was from the general community or a university student convenience sample; (b) whether there was a significant difference in baseline depression between intervention and control groups; (c) whether participants were required to meet a depression level threshold; (d) whether non-compliers were included in the analysis; (e) whether there was a high attrition rate in the intervention group between pre- and post-measurement; and (f) whether participants were compensated for being part of the study. Any criteria that were missing from a particular study were coded as ‘not reported’.

### 2.8. Statistical Methods

We performed the data analysis using Comprehensive Meta-Analysis Software Version 4 (Borenstein, 2022). Effect size was calculated using Hedges’ *g*, which is the standardised mean difference corrected for bias in small samples (Hedges, 1981). Hedges’ *g* was used because it is the most accurate effect size estimate when equal variance assumptions are violated (Marfo & Okyere, 2019). A random-effects model was used to calculate effect size as heterogeneity in sample characteristics and study design likely produces variance in true effect size between studies (Borenstein et al., 2010). Heterogeneity of effect sizes was evaluated using Cochran’s *Q* test, the *I*^2^ statistic and the tau^2^ estimate of variance (Higgins, 2008). We performed a one-study-removed analysis to assess if removing any single study had a significant influence on the total weighted effect size. We used Egger’s test for funnel plot asymmetry (Egger et al., 1997) and Duval and Tweedie’s trim-and-fill method (Duval & Tweedie, 2000) to test for publication bias. We used multivariate meta-regression and sub-group analyses to test for significant moderator effects.

## 3. Results

### 3.1. Search

**Figure 1:**
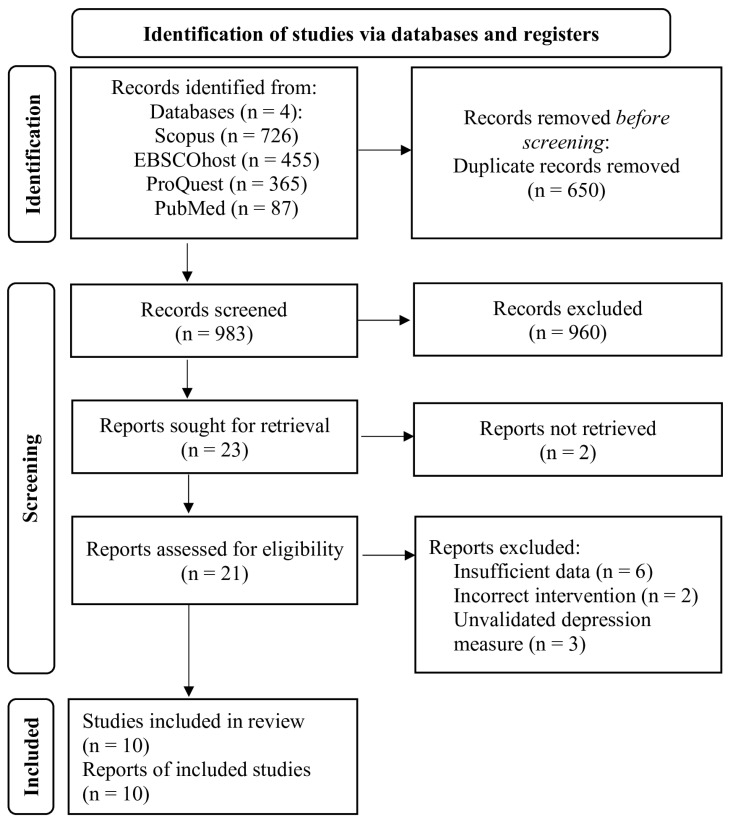
PRISMA Flow Diagram of Study Selection.

The study selection PRISMA chart is presented in Figure 1. The study search yielded a total of 1643 results. A total of 1610 results were deemed ineligible for inclusion after the removal of duplicates and initial screening of titles and abstracts. The remaining 23 articles were obtained for full-text assessment. Thirteen studies were excluded after full-text assessment, and 10 studies were included in the final analysis. Six of the thirteen excluded studies did not publish sufficient data to be included in the analysis. Reed et al. (2023), mentioned above, was one of those. Authors of these studies were contacted but did not reply to requests for further information.

### 3.2. Study Characteristics

Table 1 shows the key characteristics of included studies. Across the 10 studies, there were 1491 participants (75.39% female) with a mean age of 24.2.

### 3.3. Inclusion of Non-Compliers in Analysis and Overall Assessment of Study-Method Bias

Table 2 presents the results of a comparison of studies that included non-compliers and studied that did not. Table 3 presents the results of an assessment of risk of study-method bias involving whether non-compliers were included in the analysis.

### 3.4. Meta-Analysis Results

**Figure 2:**
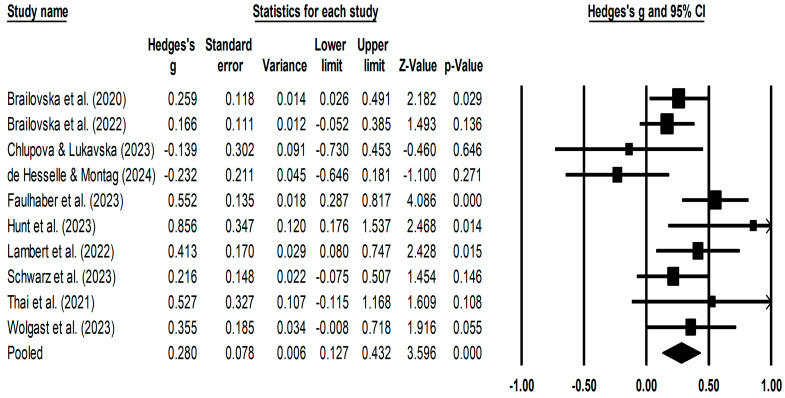
Forest Plot of Effect Sizes for Interventions That Reduce Social Media Use (Brailovskaia et al., 2020, 2022; Chlupová & Lukavská, 2023; de Hesselle & Montag, 2024; Hunt et al., 2023; Schwarz et al., 2023; Lambert et al., 2022; Thai et al., 2021; Wolgast et al., 2023).

The random-effects model showed that reducing social media use significantly reduced the severity of depressive symptoms, overall effect size *g* = 0.28, 95% CI [0.13, 0.43], *k* = 10, *p* < 0.001. Figure 2 shows the forest plot of the effect reducing social media use had on depression symptoms in each study. Heterogeneity analysis found that there was significant heterogeneity in effect sizes in the included studies (*Q* (9) = 17.24, *p* = 0.045, *I*^2^ = 47.29%, tau^2^ = 0.026).

### 3.5. Sensitivity and Publication Bias Analyses

The sensitivity analysis using the one-study-removed method showed that removing any single study from the meta-analysis produced a weighted effect size between *g* = 0.24 and *g* = 0.32. These values fall within the 95% confidence interval of the total pooled weighted effect size [0.13, 0.43]. Therefore, no single study would be considered an outlier with significant influence on the results of the meta-analysis.

**Figure 3:**
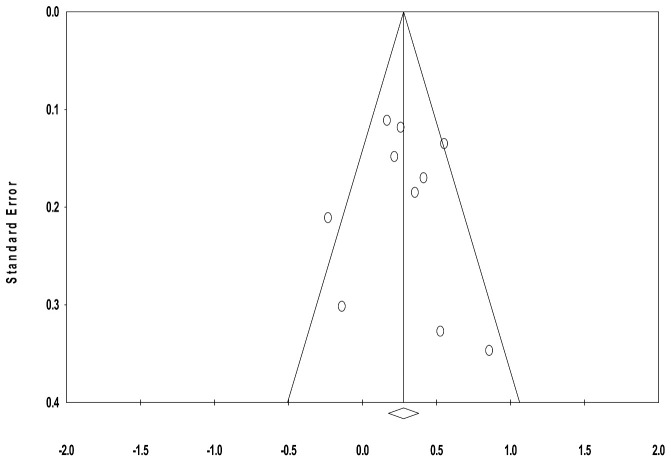
Funnel plot of standard error by Hedges’ *g*.

Figure 3 shows the funnel plot of effect sizes for the 10 included studies. Visual inspection of the funnel plot indicated symmetry, and Egger’s test for asymmetry of the funnel plot was non-significant (*p* = 0.85). Both results suggested the absence of publication bias.

Duval and Tweedie’s trim and fill indicated that one study would be imputed to adjust for potential publication bias. This adjustment decreased the estimated effect size of the intervention from *g* = 0.28 to 0.25 (95% CI [0.10, 0.41]). The reduction in the effect size after adjustment indicates that publication bias may have had a small impact on the results of the meta-analysis.

### 3.6. Moderator Analysis

The effect size of limited use interventions did not significantly differ from the effect size of full abstinence interventions (*Q* (1) = 0.85, *p* = 0.36). However, a sub-group analyses of intervention type found that only the effect size for limited use interventions was significant. Table 4 presents the results of the subgroup analyses that compared the effect of full abstinence and limited use interventions.

Table 5 shows the output of the multivariate meta-regression of continuous moderators used to assess whether effect size was moderated by the length of the intervention or number of social networking sites targeted. The length of intervention was a nonsignificant moderator of effect. The number of social networks targeted by an intervention was also a nonsignificant moderator of effect.

## 4. Discussion

This meta-analysis investigated the effects on depression of interventions that ask participants to reduce their social media use. The hypothesis that interventions reducing social media use would be significantly more effective than a neutral comparison group was supported (*g* = 0.28, *p* < 0.001). According to Cohen’s (1988) guidelines for interpretation, reducing social media use had a small effect on symptoms of depression. Significant heterogeneity was found between studies, with I^2^, indicating that 47.29% of the variability in effect sizes across studies was due to heterogeneity of true effect size rather than random error (Huedo-Medina et al., 2006). Sensitivity analyses found no individual study exerted undue influence on the overall results. Duval and Tweedie’s trim and fill method suggested imputing an additional study to adjust the effect size for publication bias. This change produced an adjusted meta-analytic effect size that still supported the main hypothesis of an effect on depression (*g* = 0.25, 95% CI [0.10, 0.41]).

Moderator analyses found no significant moderators of effect, despite the heterogeneity of effect sizes. The hypotheses that longer interventions and interventions that targeted more social networks would be more effective were not supported. The hypothesis that limited-use interventions would be more effective than full-abstinence interventions in reducing depressive symptoms was also not supported. Sub-group analysis found that the full abstinence interventions had a nonsignificant effect on depression. However, the pooled effect size for limited-use interventions was significant (*g* = 0.33, *p* < 0.001, *k* = 7). Other possible moderators of effect size were not examined in the meta-analysis because they typically were not examined in the included studies. These others include the baseline level of social media use, effect mechanisms targeted by the intervention, and what activities replaced use of social media.

The main finding of this meta-analysis was that limiting social media use significantly reduced level of depression. This result is consistent with the conclusions of previous reviews (Plackett et al., 2023; Ramadhan et al., 2024). The systematic review by Plackett et al. (2023) did not calculate an effect size but concluded that most interventions significantly reduced symptoms of depression. The meta-analysis of Ramadhan et al. included only three studies of depression effects, and one of those had an important error. The present findings provide an effect size based on 10 studies and a comparison of reduced use versus abstinence. The current finding that limiting social media use significantly reduces depression is consistent with a large body of observational research that reports time spent on social media was significantly correlated with depression (e.g., Cunningham et al., 2021; Ghai et al., 2023).

This meta-analysis was the first study to provide a robust estimate of the effect of reducing social media use on symptoms of depression, by including numerous studies with sufficient internal validity. The results demonstrate that limiting social media has a significant effect on depression across methods, measures, and research groups.

A valuable finding was that full abstinence is not required to reduce symptoms of depression and that only limiting the time one spends using social media can still produce a significant effect. This is useful information for any individuals who would struggle to completely remove themselves from social media for longer periods. Schwarz et al. (2023) found that participants struggled to fully abstain from Instagram for one-week. They reported that participants in the experimental group were significantly more likely to drop-out of the study than participants in the control group *F*(2,572) = 3.83, *p* = 0.02, η^2^ = 0.013. It could be that limiting use rather than stopping it minimized fear of losing out and reduced the stress of self-control, while still reducing social comparison.

## 5. Conclusions

The results suggest that reducing social media involvement reduces depression. More RCTs with good research methods would be useful for evaluating the depression effect with different populations.

## 6. Limitations

The quality assessment identified multiple potential threats to internal validity that limit the generalisability of the present meta-analysis. It indicated that only four out of 10 studies included data from non-compliers in their analysis. RCTs that include non-compliers measure the effectiveness of offering the intervention rather than just the intervention itself. Excluding non-compliers undermines randomisation effects by violating the assumption that the probability a participant engages with the intervention is random for all predictors of the outcome (Hewitt et al., 2006). The quality analysis also found that half the included studies did not report attrition rates. Researchers have found that bias is likely introduced when the attrition rate of an RCT is more than 20% (Moher et al., 2001). No studies that reported attrition surpassed this number; however, it is possible that the data from RCTs which did not report attrition was impacted by bias. Another important finding of the quality assessment was that nine out of 10 studies did not require participants to meet a baseline threshold for level of depression. The one study that did require a showing of depression to enter the study (Hunt et al., 2023) showed the largest effect size of all the studies, *g* = 0.86. Individuals with differences in baseline depression can often be affected in different ways by interventions targeting depression (Hengartner, 2019). Study design and the reliability and validity of depression measures were not included in the quality assessment as they were criteria for inclusion in the meta; all included studies were RCTs and used reliable, valid measure of depression. Despite the findings of the quality assessment, the strict eligibility criteria for studies in this meta-analysis protected against many threats to internal validity.

The meta-analysis did not include enough studies to evaluate whether interventions to reduce or to eliminate use of social media had more positive effects on depression in some populations versus others. Hence, the best target for the interventions remains unknow.

The small number of RCTs that have investigated the effect of reducing or refraining from social media use on depression limited the findings of this meta-analysis in multiple ways. Because the meta-analysis did not have a large number of included studies, it did not have much statistical power to identify significant moderators of effect.

None of the included RCTs investigated the effect interventions had on problematic users. The lack of studies on problematic users limits the generalisability of results to these potentially vulnerable populations. The external validity of the results was also limited by a lack of long-term follow-up data. No intervention was longer than three weeks and only three studies measured depression later than post-intervention.

## 7. Future Research

Future researchers could continue to investigate how different elements of intervention affect outcomes, focusing on limiting use rather than ending use. Focus would best be placed on investigating *how* interventions affect those who have a problematic relationship with social media or suffer from a depressive disorder. Future research could investigate whether the length of the intervention affects depression and whether the effects of these interventions on depression symptoms persist after long-term follow-up. Analysis with all participants, along with reporting of attrition level, would be appropriate.

Future interventions might aim to reduce use of social media along with efforts to reduce the amount of social comparison. By allowing participants to continue to engage with social media at a reduced level, the autonomy allowed by the interventions might have the greatest long-term impact. The interventions might also aim to not just decrease deleterious use of social media but also to promote using the newly available time for adaptive purposes.

Future research could address whether changing the nature of use of social media has a positive effect on depression. It could be that changing the nature of the use, including which sites are accessed and whether social comparisons are made, might have a positive effect on depression (see Seabrook et al., 2016). Further, it could be instructive to assess as possible mediators proposed mechanisms of change. For instance, what do participants do with their time if not engaging with social media? The research findings of Jiang et al. (2025) show that online activities can have a positive effect involving informal learning.

## Figures and Tables

**Table 1:** Key Characteristics of Included Studies.

Study Name (Author)	N	Mean Age	% Female	Country	Intervention Type	Intervention Length (Days)	Baseline to Final (Days)	Number of SM Targeted
Brailovskaia et al. (2020)	286	24.75	77.8	Germany	Limited use	14	106	1
Brailovskaia et al. (2022)	322	25.76	76.78	Germany	Limited use	14	198	All
Chlupová and Lukavská (2023)	48	21.96	72.92	Czechia	Limited use	21	21	1
Faulhaber et al. (2023)	230	22	73	US	Limited use	14	14	3
de Hesselle and Montag (2024)	90	23.750	85.56	Germany	Full abstinence	14	28	5
Hunt et al. (2023)	35	N/R	N/R	US	Limited use	21	21	5
Lambert et al. (2022)	140	28.93	61.69	UK	Full abstinence	7	7	4
Schwarz et al. (2023)	185	21.87	80	Germany	Full abstinence	7	7	1
Thai et al. (2021)	38	18	67	Canada	Limited use	21	21	7
Wolgast et al. (2023)	117	N/R	N/R	Sweden	Limited use	21	21	5

Note. N = sample size; % Female = percentage of females in sample; Country = Country that experiment was conducted; Number of SM Targeted = Number of social media platforms targeted by the intervention; SM Targeted = Which social networking sites were targeted by the intervention; Baseline to final (days) = baseline to final assessment duration in days; N/R = Not reported.

**Table 2:** Effect Size of Studies That Included Non-Compliers in Data-Analysis vs. Those That Did Not.

Were Non-Compliers Included in Final Analysis?	*k*	Hedges’ *g*	*SE*	95% CI	*p*
No	6	0.33	0.07	0.19, 0.47	<0.001
Yes	4	0.25	0.20	−0.14, 0.64	0.205

**Table 3:** Assessment of Study-Method Bias.

Study Author (Year)	Measure of Depression	Sample Type	Were Differences in Baseline Depression Between-Groups Significant?	Were Non-Compliers Included in Analysis?	Were Participants Required to Meet a Depression Threshold?	Attrition Rate of the Intervention Group Between Pre- and Post- Measurement	Were Participants Compensated for Being Part of the Experiment?
Brailovskaia et al. (2020)	DASS-21	Commun	No	Yes	No	Not reported	Yes
Brailovskaia et al. (2022)	DASS-21	Commun	No	No	No	Not reported	Yes
Chlupová and Lukavská (2023)	BDI-II	Commun	No	Yes	No	4.17%	No
Faulhaber et al. (2023)	CES-D	University	No	No	No	Not reported	Yes
de Hesselle and Montag (2024)	PHQ-2-D	Commun	No	Yes	No	15.25%	Yes
Hunt et al. (2023)	BDI-II	University	No	No	Yes	Not reported	Yes
Lambert et al. (2022)	PHQ-8-D	Commun	No	Yes	No	8.64%	No
Schwarz et al. (2023)	CES-D	University	No	No	No	Not reported	No
Thai et al. (2021)	CES-D	University	No	No	No	20%	Yes
Wolgast et al. (2023)	DASS-21	University	No	No	No	13.24%	No

Note. Sample Type = Whether a study sampled the general community (Community) or from a university student convenience sample (university); CES-D = Center for Epidemiologic Studies Depression Scale (Radloff, 1977); DASS-21 = Depression Anxiety Stress Scales—21 items; BDI-II = Beck Depression Inventory (Beck et al., 1996)—Second Edition; PHQ-2 = Patient Health Questionnaire—2 depression items (Kroenke et al., 2001); PHQ-8 = Patient Health Questionnaire—8 Depression (Kroenke et al., 2001). Commun = Community.

**Table 4:** Categorical Moderator Subgroup Analysis of Intervention Type.

Intervention Type	*k*	Hedges’ *g*	*SE*	95% CI	*p*
Limited use	7	0.33	0.09	0.16, 0.51	<0.001
Full abstinence	3	0.15	0.17	−0.18, 0.49	0.369

**Table 5:** Multivariate Meta-Regression of Continuous Moderators.

Covariate	Coefficient	*SE*	95% CI	*p* (Two-Tailed)
Intervention length (days)	0.004	0.020	−0.34, 0.04	0.84
Number of social networks targeted	0.008	0.044	−0.08, 0.09	0.85

## Data Availability

The data file is openly available at https://doi.org/10.17605/OSF.IO/Q7JFV.
